# Supplementary material for: Conditional chemoconnectomics (cCCTomics) as a strategy for efficient and conditional targeting of chemical transmission
Source: eLife. 2024 Apr 30;12:RP91927. doi: 10.7554/eLife.91927 (PMC11060718; doi:10.7554/eLife.91927)
Supplement: Figure 3—source data 3. [file elife-91927-fig3-data3.zip › Figure 3 source data3 part 2.zip!/Figure 3-Source data 3-part2/1053f2-1-CRISPResso_Report_y3hlioj1.zip!/CRISPResso_on_1053f2_2/1c.Alignment_barplot.pdf]

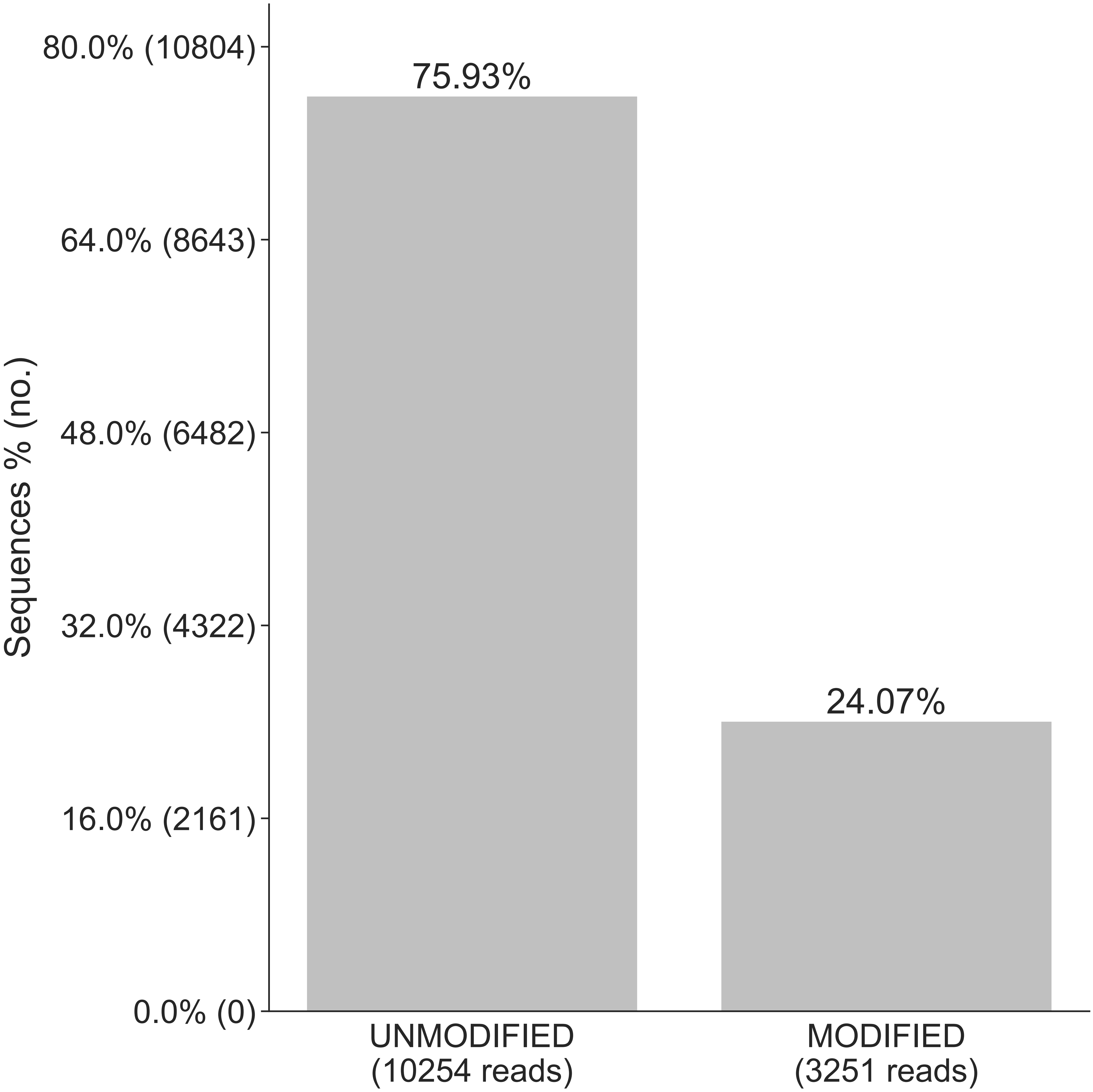

Sequences % (no.)
80.0% (10804)
64.0% (8643)
48.0% (6482)
32.0% (4322)
16.0% (2161)
0.0% (0)
75.93%
24.07%
UNMODIFIED
(10254 reads)
MODIFIED
(3251 reads)
